# Awareness of Secondary School Students regarding Basic Life Support in Abha City, Southern Saudi Arabia: A Cross-Sectional Survey

**DOI:** 10.1155/2021/4878305

**Published:** 2021-01-29

**Authors:** Bandar Almojarthe, Saad Alqahtani, Belgith AlGouzi, Wael Alluhayb, Nouf Asiri

**Affiliations:** ^1^Family Medicine Department at King Khalid University, Abha, Saudi Arabia; ^2^King Khalid University, College of Medicine, Abha, Saudi Arabia

## Abstract

**Background:**

Basic life support (BLS) is a level of medical care that is used for individuals with life-threatening illnesses or injuries until they can be given full medical care at a hospital. It can be provided by trained medical personnel, including emergency medical technicians and paramedics, and by qualified bystanders. Vital areas of adult BLS include immediate identification of sudden cardiac arrest and activation of the emergency response system, early performance of high-quality cardiopulmonary resuscitation (CPR), and rapid defibrillation, when appropriate.

**Aim:**

To assess the awareness of secondary school students regarding BLS in Abha City, Saudi Arabia. *Methodology*. A descriptive cross-sectional survey was conducted targeting all accessible secondary school students in Abha City during the academic years 2018-2019. After explaining the objectives and importance of the research topic, all students in the three grades were invited to complete the study questionnaire. The questionnaire was developed by the researchers after reviewing the literature for related topics and consulting an expert for any additions or modifications.

**Results:**

The study included 761 students with ages ranging from 15 to 20 years and a mean age of 17 ± 1 years old. Male students accounted for 53.6% of the participants, and 96.7% of the participants were Saudi. Exactly 31% of the students had had a BLS training course, among which 79.2% had had training that lasted for only one day. Regarding awareness, 65% of the students had heard about BLS, and 44% knew about CPR. Exactly 52% of the students indicated that they should call the ER if there was a case with fainting. A total of 45.3% of the students reported that airway checking was the first step in CPR, and 16.7% reported that the chest compression to oral breathing ratio should be 30 to 2. *Conclusions and Recommendations*. In conclusion, the study revealed that poor awareness regarding BLS was present among the students. The researchers concluded that less than one-third of the students had BLS training. BLS should be taught, theoretically and practically (with simulations), to middle and high school students as BLS involves relatively simple concepts and methods.

## 1. Background

Basic life support (BLS) is a level of medical care that is used for individuals with life-threatening illnesses or injuries until they can be given full medical care at a hospital. It can be provided by trained medical personnel, including emergency medical technicians and paramedics, and by qualified bystanders [1]. In 2005, the International Liaison Committee on Resuscitation (ILCOR) published the International Consensus on Cardiopulmonary Resuscitation (CPR) and Emergency Cardiovascular Care (ECC) Science with Treatment Recommendations [2]. Since 2010, the committee has provided materials for regional resuscitation providers such as the European Resuscitation Council and American Heart Association to write their own guidelines [3]. Basic life support emergency medical services in the United States are generally identified with emergency medical technician-basic (EMT-B) training [2]. However, the American Heart Association's BLS protocol is designed for use by laypeople as well as students and other certified first responders and, to some extent, higher levels of medical personnel. It includes protocols for cardiac arrest, respiratory arrest, drowning, and foreign body airway obstruction (FBAO or choking). EMT-B is the highest level of healthcare provider that is limited to the BLS protocol; higher levels of medical personnel use some or all of the advanced cardiac life support (ACLS) protocols, in addition to BLS protocols [2, 4].

BLS procedures include chest compressions, bleeding control, artificial ventilation, and basic airway management [5, 6]. Ideally, everyone should know BLS and CPR as life-threatening emergencies can occur anytime, anywhere, and to anyone. Awareness of BLS and CPR is prerequisite to acquiring the pivotal knowledge and skills to perform them correctly [7–9].

Students in secondary school are approximately 12–15 years old and have high activity levels and interaction rates due to their energy and curious nature, making them at risk for accidents, choking, or even fainting due to overexertion [10]. Their awareness regarding the BLS technique could save the lives of many of their peers even before they receive traditional medical care. The current study aimed to assess the awareness level and experience of secondary school students in Abha City regarding BLS.

## 2. Methodology

A descriptive cross-sectional survey was conducted targeting all accessible secondary school students in Abha City during the academic years 2018-2019. Abha City was divided into sectors, and within each sector, the schools were stratified according to sex (male and female). The largest male and female schools within each sector were included in the study. After explaining the objectives and importance of the research topic, all students in the three grades were invited to complete the study questionnaire. The questionnaire was developed by the researchers after reviewing the literature for related topics and consulting an expert for any additions or modifications. The questionnaire included students' biodemographic data, including their parents' education and work. The second section covered their history of training regarding BLS and the training duration. Awareness was assessed in the third section, which covered having heard about BLS, CPR, how to perform them, their steps, and their mechanisms.

## 3. Data Analysis

After the data were extracted, they were revised, coded, and input into the statistical software IBM SPSS version 22 (SPSS, Inc., Chicago, IL). All statistical analyses were performed using a two-tailed test. A *P* value less than 0.05 was considered to be statistically significant. For awareness items, each correct answer was scored one point, and the sum total of the discrete scores of the different items was calculated. A student with a score less than 50% of the maximum score was considered to have poor awareness, while good awareness was considered if the student had a score of 50% of the maximum or more. A descriptive analysis based on frequency and percent distributions was performed for all variables, including demographic data, training data, and awareness. Univariate relations between students' biodemographics and their awareness level were determined based on the Pearson chi-square test.

## 4. Results

The study included 761 students with ages ranging from 15 to 20 years and a mean age of 17 ± 1. Male students accounted for 53.6% of the participants, and 96.7% of participants were Saudi. A university education was recorded for 52.2% of the students' fathers and for 41.5% of their mothers. Approximately 63% of the students' fathers were working, and 36.7% of their mothers were also working. Exactly 54.9% of the students' families had a monthly income of 10000 Saudi riyal (1 riyal = 0.27 US dollars = 0.23 euros = 0.21 British pound) or more (Table 1).

With regard to students' training experience in BLS, Table 2 demonstrates that 31% of the students had had a BLS training course, among which 79.2% had participated in a course that lasted for only one day. The training was conducted more than a year prior to the survey among 69.9% of the trained students. Exactly 67.8% of the students took the course for their own benefit, while 16.9% completed the training for promotion (obligatory). Only 10.6% of the students felt that they had enough information regarding BLS, and 40.2% agreed that training should be obligatory for secondary school students.

In terms of awareness regarding BLS, 276 (36.3%) of the students had a good awareness level overall. Exactly 65% of the students had heard about BLS, and 44% knew about CPR. Exactly 52% of the students responded that they should call the ER if there was a case with fainting. A total of 45.3% of the students responded that airway checking was the first step in CPR, and 16.7% responded that the chest compression to oral breathing ratio should be 30 to 2 (Table 3).

Regarding relating students' awareness to their personal data (Table 4), it was clear that 41.1% of students with highly educated fathers had good awareness levels, compared to 21.2% of students with illiterate fathers. This difference was found to be statistically significant (*P*=0.011). Additionally, 41.8% of students with highly educated mothers had a good awareness level, compared to 23.3% of students with illiterate mothers (*P*=0.010). In terms of training, 51.3% of the students who had training regarding BLS reported a high awareness level in our total survey score compared to 29.5% of those who did not (*P*=0.001). Additionally, 46.4% of those who had faced a case requiring CPR had a good awareness level, compared to 33.8% of those who did not (*P*=0.004).

**Figure 1:**
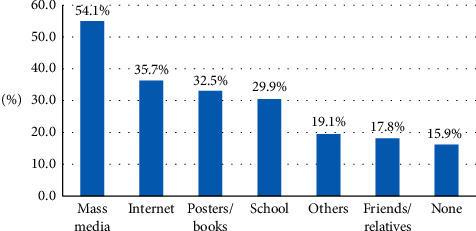
Source of BLS information reported by secondary school students in Abha City, southern Saudi Arabia.

Finally, mass media was the most frequently reported source of student information regarding BLS (54.1%), followed by Internet (35.7%) and posters and books (32.5%), while 15.9% of the students reported no specific source (Figure 1).

## 5. Discussion

Basic life support (BLS) includes activities and services that are used to save persons from life-threatening dangers until they can receive appropriate medical care at the hospital. BLS procedures include cardiopulmonary resuscitation (CPR), bleeding control, artificial ventilation, and basic airway management [7, 11]. Cardiac arrest and road traffic accidents are the most frequent emergencies with serious outcomes. Most mortality associated with them may be prevented by vital, readily acquired, maneuvers, and skills. Cardiac and respiratory arrests are very common emergencies not only in adults but also in young people. These dangers can be easily addressed with awareness and practice of resuscitation maneuvers [12]. This is the reason for the increased demand for teaching and training of nonhealth professionals and laypersons regarding effective and safe resuscitation prior to hospital care, in order to improve the clinical outcomes of these emergencies and reduce the death rates [6, 13].

The current study aimed to assess the awareness level regarding BLS among secondary school students, which is a highly dynamic group with an increasing incidence of emergencies and trauma. Many studies have focused on the awareness of teachers, which is vital, but students' awareness will significantly help as the people nearest to the victim are likely to be his/her peers. The current study revealed that nearly one out of three students had good awareness regarding BLS. This may be explained by the fact that one-third of them had received training for BLS in the past year. The highest awareness was recorded for the first step being to call the ER in the case of fainting, while the most frequently recorded incorrect answer was regarding the chest compression to oral breathing ratio. Students' awareness increased significantly in direct relation with their parents' education, training history, and the history of experiencing a case that required resuscitation. Additionally, among the interesting findings was that medical staff had no role at all in providing students with information regarding BLS, as the main sources of knowledge were mass media, the Internet, books, and friends. Expanding the role of medical staff in educating students about BLS seems warranted.

These findings were consistent with those recorded by others in many areas. In Germany, a study revealed that only 29.5% of high school students performed chest compressions during BLS correctly [14]. A second study was conducted in Denmark and revealed that Danish high school students recorded poor awareness regarding BLS, and the majority were afraid of performing BLS [15].

Locally, a study conducted in Riyadh, including 580 secondary school students, found that 56% of them had inaccurate CPR information, and approximately 67% of all students were willing to learn more about CPR [16]. A second study was conducted in Taif, including 360 male secondary school students, to assess the awareness of secondary school students regarding first aid and BLS. The average score of the students' awareness was 64.8%, and the trained students (79.6%) reported better first aid knowledge and skills than untrained students (53.7%) [17].

Life-threatening emergencies can occur anytime, anywhere, and to anyone. The outcomes of these events can be improved via awareness and the implementation of resuscitation maneuvers.

## 6. Study Limitation

This is a small-scale descriptive cross-sectional study focusing on target population at a certain area not merely including all population. Probably, a larger study may need to be undertaken before any public health decisions are made.

## 7. Conclusions and Recommendations

In conclusion, approximately one-third of the students were aware of basic life support and CPR for emergencies. Additionally, researchers concluded that less than one-third of the students had BLS training. BLS should be taught, theoretically and practically (with simulations), to middle and high school students as BLS involves relatively simple concepts and methods. This can be in parallel with national campaigns for improving community awareness and skills.

## Figures and Tables

**Table 1:** Biodemographic data of secondary school students in Abha City, southern Saudi Arabia.

Biodemographic data		No.	%
Age in years	15-16	255	33.5
	17-18	473	62.2
	19-20	33	4.3

Sex	Male	408	53.6
	Female	353	46.4

Education level	First grade	277	36.4
	Second grade	187	24.6
	Third grade	297	39.0

Nationality	Saudi	736	96.7
	Non-Saudi	25	3.3

Father's education	Illiterate	33	4.3
	Basic	141	18.5
	Intermediate	0	0.0
	Secondary	190	25.0
	University/above	397	52.2

Mother's education	Illiterate	90	11.8
	Basic	168	22.1
	Intermediate	0	0.0
	Secondary	187	24.6
	University/above	316	41.5

Father's work status	Yes	480	63.1
	No	86	11.3
	Retired	195	25.6

Mother's work status	Yes	279	36.7
	No	467	61.4
	Retired	15	2.0

Monthly income	<5000 SR	129	17.0
	5000–9000 SR	214	28.1
	10000–20000 SR	240	31.5
	>20000 SR	178	23.4

**Table 2:** BLS training history among secondary school students in Abha City, southern Saudi Arabia.

BLS training history		No.	%
Had training for BLS	Yes	236	31.0
	No	525	69.0

If yes, duration (*n* = 236)	One day	187	79.2
	2–5 days	23	9.7
	Week	15	6.4
	More	11	4.7

Duration since training	1 year ago	71	30.1
	Less than 1 year	102	43.2
	More than 1 year	63	26.7

Motive to undergo the training (*n* = 236)	Obligatory	40	16.9
	For benefit	160	67.8
	Previous case	23	9.7
	Others	13	5.5

Feeling of having enough information regarding BLS	Yes	81	10.6
	No	680	89.4

Agree regarding obligatory BLS training for secondary school students	Obligatory	306	40.2
	Elective	326	42.8
	No need	129	17.0

**Table 3:** BLS awareness recorded among secondary school students in Abha City, southern Saudi Arabia.

BLS awareness data		No.	%
Know about BLS	Yes	495	65.0
	No	266	35.0

First aid for fainting	Call ER	396	52.0
	Start CPR	269	35.3
	Go to nearest hospital	96	12.6

Know what CPR is	Yes	335	44.0
	No	426	56.0

First step of resuscitation	Chest compressions	208	27.3
	Airway checking	345	45.3
	Oral breathing	50	6.6
	Do not know	158	20.8

Chest pressure to oral breathing ratio	15 : 2	156	20.5
	30 : 1	73	9.6
	15 : 1	49	6.4
	30 : 2	127	16.7
	Do not know	356	46.8

**Table 4:** BLS awareness of secondary school students in Abha City, southern Saudi Arabia, by demographics.

Personal data		Awareness level				*P* value
		Poor		Good		
		No.	%	No.	%	
Age in years	15-16	167	65.5	88	34.5	0.541
	17-18	295	62.4	178	37.6	
	19-20	23	69.7	10	30.3	

Sex	Male	260	63.7	148	36.3	0.997
	Female	225	63.7	128	36.3	

Education level	First grade	175	63.2	102	36.8	0.798
	Second grade	123	65.8	64	34.2	
	Third grade	187	63.0	110	37.0	

Father's education	Illiterate	26	78.8	7	21.2	0.011^*∗*^
	Basic	101	71.6	40	28.4	
	Secondary	124	65.3	66	34.7	
	University/above	234	58.9	163	41.1	

Mother's education	Illiterate	69	76.7	21	23.3	0.010^*∗*^
	Basic	112	66.7	56	33.3	
	Secondary	120	64.2	67	35.8	
	University/above	184	58.2	132	41.8	

Had training for BLS	Yes	115	48.7	121	51.3	0.001^*∗*^
	No	370	70.5	155	29.5	

Duration since training	1 year ago	37	52.1	34	47.9	0.196
	Less than 1 year	43	42.2	59	57.8	
	More than 1 year	35	55.6	28	44.4	

Faced a case needing CPR	Yes	81	53.6	70	46.4	0.004^*∗*^
	No	404	66.2	206	33.8	

*P*, Pearson *X*^2^ test. ^*∗*^*P* < 0.05 (significant).

## Data Availability

The data used to support the findings of the study are available from the corresponding author upon request.
